# Phenolic Compounds Isolated from Olive Oil as Nutraceutical Tools for the Prevention and Management of Cancer and Cardiovascular Diseases

**DOI:** 10.3390/ijms19082305

**Published:** 2018-08-06

**Authors:** Patricia Reboredo-Rodríguez, Alfonso Varela-López, Tamara Y. Forbes-Hernández, Massimiliano Gasparrini, Sadia Afrin, Danila Cianciosi, Jiaojiao Zhang, Piera Pia Manna, Stefano Bompadre, José L. Quiles, Maurizio Battino, Francesca Giampieri

**Affiliations:** 1 Dipartimento di Scienze Cliniche Specialistiche ed Odontostomatologiche (DISCO)-Sez. Biochimica, Facoltà di Medicina, Università Politecnica delle Marche, 60131 Ancona, Italy; preboredo@uvigo.es (P.R.-R.); avarelalopez@gmail.com (A.V.-L.); tamara.forbe@gmail.com (T.Y.F.-H.); m.gasparrini@univpm.it (M.G.); dolla.bihs@gmail.com (S.A.); danila.cianciosi@gmail.com (D.C.); zh.jojo@yahoo.com (J.Z.); p.piera@hotmail.it (P.P.M.); 2 Departamento de Química Analítica y Alimentaria, Grupo de Nutrición y Bromatología, Universidade de Vigo, 32004 Ourense, Spain; 3 Dipartimento di Scienze Biomediche e Sanità Pubblica, Università Politecnica delle Marche, 60131 Ancona, Italy; s.bompadre@univpm.it; 4 Department of Physiology, Institute of Nutrition and Food Technology “José Mataix”, Biomedical Research Center, University of Granada, Avda del Conocimiento sn., 18100 Armilla, Granada, Spain; jlquiles@ugr.es

**Keywords:** olive oil, bioactive compounds, cancer, cardiovascular diseases, prevention, Mediterranean Diet

## Abstract

Non-communicable diseases (NCDs) have become the largest contributor to worldwide morbidity and mortality. Among them, cancer and cardiovascular diseases (CVDs) are responsible for a 47% of worldwide mortality. In general, preventive approaches modifying lifestyle are more cost-effective than treatments after disease onset. In this sense, a healthy diet could help a range of NCDs, such as cancer and CVDs. Traditional Mediterranean Diet (MD) is associated by the low-prevalence of certain types of cancers and CVDs, where olive oil plays an important role. In fact, different epidemiological studies suggest that olive oil consumption prevents some cancers, as well as coronary heart diseases and stroke incidence and mortality. Historically, the beneficial health effects of virgin olive oil (VOO) intake were first attributed to the high concentration of monounsaturated fatty acids. Nowadays, many studies indicate that phenolic compounds contained in olive oil have positive effects on different biomarkers related to health. Among them, phenolic compounds would be partially responsible for health benefits. The present work aims to explore, in studies published during the last five years, the effects of the main phenolic compounds isolated from olive oil on different cancer or CVD aspects, in order to clarify which compounds have more potential to be used as nutraceuticals with preventive or even therapeutic properties.

## 1. Introduction

Nowadays, Non-communicable diseases (NCDs) have become the largest contributor to worldwide morbidity and mortality even in low- and middle-income countries, causing 17 million of premature deaths (under the age of 70) [1]. Among them, cancer and cardiovascular diseases (CVDs) are responsible for a 47% of worldwide mortality. In 2015, overall age-standardized cancer incidence rates were 205 and 165 cases per 100,000 person-years in men and women, respectively, being responsible for a 16% of global deaths [1]. Regarding the type of cancer or organ affected, the five most common types of cancer causing death in 2015 were (in the order of frequency) lung, liver, stomach, colorectal and prostate cancers in men, whereas in women were breast, lung, colorectal, cervical and stomach cancers [1]. In addition, economic impact of cancer is significant and is increasing. It has been estimated that the total annual economic cost of cancer was approximately US$ 2.5 trillion in 2015 [2].

Notwithstanding, the first cause of death globally are CVDs, a group of disorders affecting heart and blood vessels that represent 31% of all global deaths in 2015 [1] including coronary heart disease (CHD), cerebrovascular disease, peripheral arterial disease, pulmonary embolism, rheumatic heart disease, and congenital heart disease and deep vein thrombosis. All these disorders are commonly caused by build-up of fatty deposits on the inner walls of the blood vessels that supply the heart, brain, or even arms and legs, causing a blockage that prevents blood from flowing to these organs. Strokes can also be caused by bleeding from a blood vessel in the brain or from blood clots. Among CVD-caused deaths, an estimated 7.4 million were due to CHD and 6.7 million were due to strokes. In addition, the risk of suffering this event is increased by other disorders that could be named as “intermediate risks factors”, such as hyperlipidemia, hypertension, diabetes, overweight and obesity. CVDs place a heavy burden on the economies of low- and middle-income countries, but they also contribute to poverty at the household level due to catastrophic health spending and high out-of-pocket expenditure.

It has been estimated that between 30 and 50% of cancers [3], as well as most CVDs, are largest preventable by healthy lifestyle choices. Both pathologies share behavioral risk factors, such as tobacco use, unhealthy diet and obesity, physical inactivity and harmful use of alcohol that could be addressed using population-wide strategies to reduce mortality and morbidity associated [4,5]. Changes in these behavioral risk factors may lead to the improvement of intermediate risk factors (blood pressure, blood glucose, blood lipids, overweight and obesity). In general, preventive approaches to modifying lifestyle are more cost-effective than treatments after disease appears. Therefore, consuming a healthy diet throughout the life course can help to prevent a range of NCDs, such as cancer and CVDs. Although healthy diet features depend on individual features (e.g., age, gender and lifestyle), available foods, dietary customs, and even cultural context, some basic principles of what constitute a healthy diet have been established. During the adult stage, a healthy diet should contain vegetables, fruits, legumes, whole grains and nuts [6], less than 10% and 30% of total energy intake from free sugars and fats, respectively [6,7,8], as well as less than 5 g of salt per day. Concerning fat, unsaturated fats are preferable to saturated fats [7] and trans-fats should be reduced to less than 1% of total energy intake [6,7]. This is supported by the low-prevalence found for some NCDs including certain types of cancers and CVDs in countries from the Mediterranean region in comparison to the other parts of the world [9,10,11,12,13,14]. A similar association of adherence to a Mediterranean Diet (MD) with cancer mortality, risk of several cancer types [15], and CVD incidence and its associated mortality have been reported [15,16].

## 2. Olive Oil and Phenolic Compounds

Traditional MD is characterized by high intake of cereals, vegetables, fruits, nuts, legumes, low intake of meat and meat products, a moderate intake of fish and seafood and a modest consumption of alcohol [17], accompanied by a regular intake of olive oil (OO), mainly virgin OO (VOO) or extra virgin OO (EVOO), of which consumption typically ranges between 25 and 50 mL (approximately two tablespoons) per day [18,19]. Actually, different authors have stated the NCDs lowered incidences to OO intake, at least in part. Different epidemiological studies suggested that OO consumption is implicated in preventing certain cancers, with the most promising findings for breast and many digestive tract cancers [12,20,21,22,23,24], as well as CHD and stroke mortality and incidence [25,26,27,28,29]. The suggested OO’s benefits on cardiovascular health and CVD risk factors are supported by strong mechanistic evidence from experimental studies [30,31,32,33].

EVOO and VOO come mostly from the mesocarp (epicarp and fleshy mesocarp, approximately 95%) with a small contribution from the seed of the fruit (endosperm and embryo, approximately 5%), after first and second pressings of the olive fruit by a cold-pressing method, where no chemicals and only a small amount of heat are applied [34,35]. OO is a complex mixture of over 200 compounds, of which composition depends on many factors, including geographical origin, weather and irrigation, ripening and processing after harvesting [36]. The main constituents of OO are triglycerides, the so-called saponifiable fraction (98–99%). The main fatty acids in the triglyceride fraction are a monounsaturated fatty acid (oleic acid, 55–83%), a saturated fatty acid (palmitic acid, 7.5–20%) and a polyunsaturated fatty acid (linoleic acid, 2.5–21%) [37,38,39]. The remaining unsaponifiable fraction (1–2%) contains (i) lipophilic phenols (tocopherols), (ii) sterols, the main sterol being beta-sitosterol, (iii) color pigments, mainly chlorophylls and carotenoids (the most important is beta-carotene), (iv) alcohols, (v) waxes, aldehydes, esters, ketones and (vi) phenolic compounds (hydrophilic phenols) [36].

Historically, the beneficial health effects of VOO intake were first attributed to the high concentration of monounsaturated fatty acids [18,35]. Oleic acid showed its ability to protect against cardiovascular events, ameliorate insulin resistance, improve endothelial dysfunction and inflammation, and reduce proliferation and apoptosis in vascular smooth muscle cells (VSMCs), compared with saturated fatty acids, such as palmitic acid. Such effects would be possibly triggered via the inhibition of JNK-1/2 and NF-κB pathways, at least in part [40]. These activities may contribute to an ameliorated atherosclerotic process and its stability in vivo. After that, studies performed thus far have demonstrated that OO phenolic compounds have positive effects on various physiological biomarkers, implicating phenolic compounds as partially responsible for health benefits associated with the MD, including anti-microbial, anti-oxidant and anti-inflammatory activities [11,13,23,41,42,43,44,45].

In VOO, the main classes of phenols are phenolic acids, phenolic alcohols, flavonoids, secoiridoids and lignans. Secoiridoids present elenolic acid (EA) or its derivative forms in their molecules and usually appear as aglycon derivatives. The most abundant secoiridoids in VOO are: (i) the dialdehydic form of decarboxymethyl-EA linked to hydroxytyrosol (HT) or tyrosol (Tyr) (3,4-DHPEA-EDA and p-HPEA-EDA, respectively), (ii) an isomer of oleuropein (OLE) aglycone (3,4-DHPEA-EA) and (iii) the ligstroside aglycone (p-HPEA-EA). These aglycone derivatives of secoiridoid glucosides are mostly originated during mechanical extraction process of oil, as a consequence of oleuropein (OLE) hydrolysis, as well as of demethyloleuropein, and ligstroside owing to endogenous β-glucosidases [37]. The absolute concentration of phenols in OO is the result of a complex interaction between several factors, including cultivar, degree of maturation, climate and other agronomic and technological factors, such as the extraction procedures [46,47,48,49,50,51,52].

Nowadays, EVOO can be promoted for its important health-promoting properties [53,54,55], mostly attributable to its content in polyphenols and well-known for their antioxidant power. In support of this, an important claim by the European Food Safety Authority (EFSA) was released in 2011, based on several scientific investigations concerning the role of phenols in human health [56]. Based on EFSA opinion, European Community Regulation 432/2012 [57] reported, among others, the health claim for OO polyphenols “*olive oil polyphenols contribute to the protection of blood lipids from oxidative stress*” that only can be used for OOs which contain at least 5 mg of HT and its derivatives (e.g., oleuropein complex and Tyr) per 20 g of OO.

Recognized properties of polyphenols from OO make this compound an optimal candidate to be used as a nutraceutical. This term has been defined by Defelice [58] as “a food or parts of food that provide medical or health benefits, including the prevention and/or treatment of disease”. Nutraceutical properties of foods have attracted attention from agri-food sector [59]. Several agricultural products have been found to naturally present healthy components in numerous studies. To date, more than 36 phenolic compounds have been isolated from EVOO and identified, although they are present at very different concentrations (0.02–600 mg/kg) [45]. Most of phenolic compounds from OO have been shown to be highly bioavailable, reinforcing their potential health-promoting properties [60,61,62].

The bioavailability of OO phenolics has been determined by measuring the concentration of the phenolic compounds and their metabolites in biological fluids, mainly plasma and urine, after ingestion of pure compounds or OO, either pure or enriched with the phenolics [63]. Research conducted on humans showed that HT and Tyr are dose-dependently absorbed after ingestion [60,64,65,66]. Vissers et al. [62] demonstrated that absorption of HT, ligstroside, oleuropein, and Tyr, was as high as 55–66%. In another study, Miro-Casas et al. [67] reported that even from moderate doses (25 mL/d), approximately 98% of HT was present in plasma and urine, mainly as glucuronide conjugates.

As a consequence of health, as well as economical and global importance of cancer and CVDs, the relationship between these diseases and OO, mainly EVOO or VOO, has become very important nowadays. The present work aims to explore the effects of the main phenolic compounds isolated from OO on different cancer or CVD aspects in studies published during the last five years with the intention to clarify which compounds have more potential to be used as nutraceuticals with preventive or even therapeutic properties. Alternatively, some of these compounds could also be added or potentiated in some food industry products yielding new functional foods.

### 2.1. Hydroxytyrosol and Derivatives

**Figure 1:**
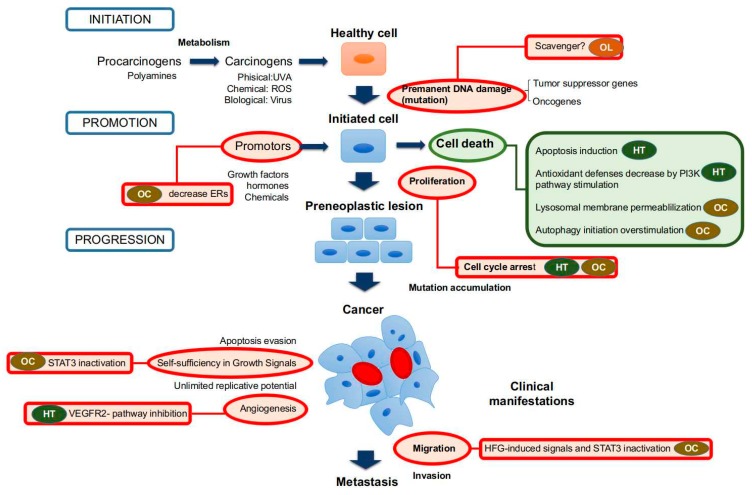
Anti-tumor and anti-cancer effects of main phenolic compounds found in olive oil (OO). Red circles mean inhibitory activities, and green circles mean stimulatory effects. Abbreviations: HT: hydroxytyrosol; OC: oleocanthal; OLE: oleuropein.

HT is a phenyl ethyl alcohol, mainly generated during storage of VOO as a consequence of hydrolytic mechanisms that release it from other complex phenols as secoiridoids [68,69]. The amount of HT that could be formed during VOO storage depends on the storage temperature, as well as on the initial concentration of the complex forms (ranging from 8.4 μmol/kg to 65 μmol/kg after 18 months at 25 °C in El Hor and Chetoui OOs, respectively) [70]. Anti-tumor activity of HT has been evidenced by several in vitro studies. In the last five years, it has been reported that treatments with HT exerted anti-proliferative effects on DLD1 human colon cells [71], TFK-1 and KMBC human cholangiocarcinoma cells and GBS-SD human gallbladder cancer cells [72] and hepatocellular carcinoma cells (HepG2, Hep3B, SK-HEP-1 and Huh-7) [73], as well as on breast (MDA and MCF-7), prostate (LNCap and PC3) and colon (SW480 and HCT116) cancer cell lines [74]. However, the magnitude of these anti-proliferative effects have been reported to depend on cell type; one of the studies on prostate cancer cells showed more resistance to growth inhibition respect than breast and colon cancer cells [74]. Anti-tumor effects of HT could also reside in its reported capacity to reduce cell viability and/or promote apoptotic cell death on human cholangiocarcinoma (TFK-1 and KMBC) [72], colon cancer (DLD1) [71], gallbladder cancer (GBS-SD) [72], hepatocellular carcinoma (HepG2, Hep3B, SK-HEP-1 and Huh-7) [73] and papillary (TPC-1, FB-2) and follicular (WRO) thyroid cancer cells [75] (Figure 1, Table 1).

Anti-proliferative effects have been associated with an arrest of cell cycle in G2/M phase (Figure 1) in TFK-1 and KMBC human cholangiocarcinoma cells, GBS-SD human gallbladder cancer cells [72] and several hepatocellular carcinoma cells (HepG2, Hep3B, SK-HEP-1 and Huh-7) [73], which resulted in cellular apoptosis in many but not all cell types [72]. In relation with these findings, Toteda et al. [75] reported a reduction of cyclin D1 expression and an up-regulation of cell cycle key modulator p21 levels in papillary (TPC-1, FB-2) and follicular (WRO) thyroid cancer cell lines, which were associated with a decreased viability and increased apoptosis markers. Finally, López De Las Hazas et al. [76] studied the anti-proliferative and apoptotic activities of HT and its main catabolites detected in human feces (phenylacetic, phenylpropionic, hydroxyphenylpropionic, and dihydroxyphenylpropionic acids and catechol) in colon cancer cell lines (Caco-2 and HT-29). The results from this study suggested HT, phenylacetic and hydroxyphenylpropionic acids are able to block cell cycles and promote apoptosis. Caco-2 cells were more resistant to phenol treatments than HT-29 cells. On the other hand, Rosignoli et al. [74] correlated anti-proliferative activities with the H_2_O_2_ accumulation in breast (MDA and MCF-7), prostate (LNCap and PC3) and colon (SW480 and HCT116) cancer cell lines. Similarly, increased intracellular reactive oxygen species ROS were found in DLD1 human colon cancer after incubation with HT [71]. This suggests that HT could cause oxidative stress in cancer cells with subsequent cellular damage that possibly leads to cell death or, at least, to cell cycle arrest. In fact, Rosignoli et al. [74] also reported that the ability of the different cell lines to remove H_2_O_2_ from the culture medium was inversely correlated with their sensitivity to the anti-proliferative effect of HT. On the other hand, ROS generated after treatments with HT could act as a secondary messenger triggering or inhibiting different cellular signaling pathways. In this sense, Zhao et al. [73] found that HT suppressed activation of Akt and nuclear factor-kappa B (NF-κB) pathways in hepatocellular carcinoma cells (HepG2, Hep3B, SK-HEP-1 and Huh-7), which could be related with its negative effects on cell cycle and proliferation. In contrast, it activated the PI3K/Akt pathway leading to FOXO3a phosphorilation and then down-regulated FOXO3a’s target genes in DLD1 human colon cancer cells promoting in this case a situation of oxidative stress [71]. HT could also negatively modulate the extracellular signal-regulated kinase (ERK) pathway, of which alteration towards over-activation has been usually considered as a necessary step in the development of many cancers, since time- and dose-dependent decrease of phosphorylated form of the ERK has been observed after HT treatments in human hepatocellular carcinoma cells [73]. Finally, HT can affect vascular endothelial growth factor (VEGF)-induced endothelial cell proliferation and migration, as well as their morphogenic differentiation into capillary-like tubular structures in Matrigel (in vitro angiogenesis), being one of the most potent angiogenesis inhibitor in a study comparing different compounds from EVOO. Such an effect would depend on inhibitory action on specific autophosphorylation sites of VEGF receptor-2 (VEGFR-2), leading to the inhibition of endothelial cell signaling [77].

In rats, HT has been found to inhibit tumor growth and proliferation rates in DMBA-induced breast cancer when it was administrated during 6 weeks after the treatment [78]. Results were comparable to those of doxorubicin, a chemotherapeutic agent commonly used in breast cancer treatment, and proved better with regard to the histopathological outcome. Then, Li et al. [72] observed anticancer effects of HT intraperitoneally administered on a cholangiocarcinoma mouse model, where HT markedly inhibited tumor growth. Likewise, tumor growth and angiogenesis results negatively affected by intraperitoneal HT in an orthotopic model of human hepatocellular carcinoma in mouse [73]. Despite the fact that the compound did not reach tumor cells by oral ingestion, some of the reported effects on tumors were similar to those noted in the DMBA-induced breast cancer model [78], and some of the mechanisms observed could also be present in animals receiving HT orally. Different mechanisms responsible for these activities have been reported in mentioned studies including the alteration of the expression of genes related to the apoptosis, cell cycle, proliferation, differentiation, survival and transformation pathways [78], which could be explained, at least in part, by the activation of Akt [73], NF-κB [73] or Wnt signaling pathway, promoting a high expression of secreted frizzled-related protein 4 [78].

Beyond anti-tumoral activities, some compounds present in EVOO could exert a preventive effect decreasing susceptibility to cancer. di Francesco et al. [79] investigated the effects of EVOO, its phenolic compounds or HT on endocannabinoid system (ECS) gene expression via epigenetic regulation in human colon cancer cells (Caco-2) exposed to dietary EVOO. They observed a selective and transient up-regulation of *CNR1* gene that was evoked by exposure of Caco-2 cells to EVOO, its phenolic extracts or HT for 24 h. None of the other major elements of the ECS was affected at any time point. The stimulatory effect of phenolic extracts and HT on cannabinoid type 1 receptor expression was inversely correlated to DNA methylation at *CNR1* promoter and was associated with reduced proliferation of Caco-2 cells.

On the other hand, it was reported the attenuation of inducible nitric oxide synthase (iNOS), cyclooxygenase-2 (COX-2) and tumor necrosis factor α (TNFα) by HT in lipopolysaccharide (LPS)-challenged human monocytic THP-1 cells [80]. It has also been reported that HT possessed significant anti-inflammatory actions in an animal model of inflammation and attenuated TNFα and interleukin 1 beta (IL-1β) expression [81]. More recently, HT at nutritionally relevant concentrations has shown to down-regulate the gene expression of adhesion molecules, chemoattractants, matrix metalloproteinases (MMPs) and proinflammatory enzymes [82,83].

Giordano et al. [84] reported that long-term supplementation with nutritionally-relevant doses of HT-modulates adipose tissue and glutathione metabolism; that is, a 24-h pretreatment with HT, followed by exposition to H_2_O_2_, maintained a higher intracellular total and oxidized glutathione (GSSG)-to-reduced glutathione (GSH) ratio compared with H_2_O_2_ alone, indicating a preventive activity against oxidative stress [84].

HT anti-inflammatory and antioxidant properties could also be very interesting for cardiovascular health, as evidenced by a recent randomized control trial, where HT, used as a functional ingredient in biscuits, has shown to lower postprandial oxidized low-density lipoprotein levels [88]. However, other studies suggested that the main HT effects are a consequence of increasing endogenous vitamin C levels, since it did not influence blood lipids, inflammatory markers, liver or kidney functions [89]. In particular, HT has been shown to beneficially impact the hemostatic profile, exerting antiplatelet and antithrombotic properties [25,91].

Concerning HT derivatives, Burattini et al. [86] investigated the effects of HT and the corresponding ester hydroxytyrosyl laurate (laur-HT) in U937 cells (a human monocytoid cell line) and in C2C12 myoblasts (a murine proliferating muscle cell model) after apoptotic death induction. The results obtained showed that laur-HyT had a protective antioxidant effect against H_2_O_2_ treatment. In an interesting view, Giordano et al. [90] synthesized two among the most physiologically prominent HT hepatic metabolites, i.e., 3-O-HT glucuronide and 4-O-HT glucuronide and tested their activities on endoplasmic reticulum (ER) stress in human hepatocarcinoma HepG2 cells. Both compounds inhibited tunicamycin-induced ER stress that can lead to unfolded protein response and ER-initiated apoptosis that have been implicated in the pathophysiology of various CVDs, including heart failure and ischemic heart [92]. An assay performed by Storniolo et al. [87] evidenced that HT and some polyphenols contained in VOO or EVOO could prevent negative consequences of less “healthy” diets rich in fat and/or sugars. It was demonstrated that treatments with HT or polyphenol extract from EVOO reverted multiple changes induced by high glucose and free fatty acids (FFAs) (features found in diabetes patients) which included nitric oxide (NO) level reduction and increased endothelin-1 (ET-1) levels induced by acetylcholine by modulating intracellular Ca^2+^ levels and endothelial nitric oxide synthase phosphorylation. This has important implications since endothelium participates in blood pressure regulation through the release of potent vasodilator and vasoconstrictor agents, such as NO and ET-1, respectively. These last findings might have implications for reducing risks of many CVDs, where the dysfunction of the endothelium is the underlying causative factor.

### 2.2. Tyrosol

Tyr is a simple phenol, namely a phenyl ethyl alcohol. As it occurs with HT, it might be released during storage of VOO by hydrolytic mechanisms from complex phenols as secoiridoids [68,69]. Tyr shows potent influences on cell proliferation, cell cycle progression, apoptosis and arachidonic acid metabolism in cancer cells [18,93,94]. Prevention of the persistent damaging effects of ROS has also been reported for HT with Tyr, either per se or in combination (each at 10 µM) (at the recommended concentration from EVOO consumption) in human umbilical cord vein endothelial (HUVEC) cells [85].

### 2.3. (−)-Oleocanthal

Oleocanthal is the term used to refer to p-HPEA-EDA [95,96] that presents two possible enantiomers. Importantly, both forms cause a dose-dependent inhibition of cyclo-oxygenase (COX)-1 and COX-2 activities comparable to that of ibuprofen but have no effect on lipoxygenasein vitro. Moreover, oleocanthal presents potent pharmacological actions in attenuating inflammatory mediators, such as iNOS [97,98]. Interestingly, oleocanthal has shown to resist hydrolysis under stomach-simulated conditions and cooperates inhibiting growth of Helicobacter pylori that has been associated with digestive system pathologies, such as gastric cancer and petic ulcer [99].

In addition to its putative anti-inflammatory potential, (−)-oleocanthal has shown anti-proliferative, but also anti-migratory activity against different cancer cell lines (Table 2). It also promotes cell apoptosis and induces DNA fragmentation in HT-29 cells, which would depend on activation of both, caspase-3 and poly-adenosine diphosphate-ribose polymerase, and phosphorylation of p53 [98]. In in vivostudies, oleocanthal inhibits proliferation and transformation of JB6 Cl41 epidermal cells from mouse, by suppressing activation of extracellular signal-regulated kinases 1/2 and p90 ribosomal S6 kinase [98]. Akl et al. [100] showed that (−)-oleocanthal treatment causes a dose-dependent inhibition of Hepatocyte Growth Factor (HGF)-induced cell migration and invasion on different human breast cancer cell lines (MDA-MB-231, MCF-7 and BT-474), along with the arrest of cell cycle at G1/S phase and cell proliferation. These effects are found to be mediated, at least in part, via the inhibition of HGF-induced c-Met activation and its downstream mitogenic signaling pathways (RAS, PI3K, beta-catenin, or STAT pathways) that are related to proliferation and surveillance of cells. In the model used, this effect would be associated with the blockade of epithelial-to-mesenchymal transition and a reduction in cellular motility. In relation with previous findings, Pei et al. [101] showed that (−)-oleocanthal inhibits human hepatocellular carcinoma tumor growth and metastasis through STAT3 inactivation. On the other hand, (−)-oleocanthal cell death induces both primary necrotic and apoptotic cell death via induction of lysosomal membrane permeabilization on human pancreatic (BxPC3), prostate (PC3) and breast (MDA-MB-231) cancer cell lines [102]. Khanfar et al. [103] also reported that docked oleocanthal inhibita the growth of several breast cancer cell lines (MCF-7 and T47D) at low micromolar concentrations in a dose-dependent manner. (−)-oleocanthal treatment also causes a marked down-regulation of phosphorylated mTOR in metastatic breast cancer cell lines (MDA-MB-231) [103], which suggests an important stimulation of autophagy initiation by this compound with negative consequences for cancer cells viability. Finally, Ayoub et al. [104] showed that (−)-oleocanthal inhibites growth of BT-474, MCF-7 and T-47D human breast cancer cells in mitogen-free media and in 17β-estradiol-supplemented media. Thus, (−)-oleocanthal can reduce total levels of estrogen receptors in BT-474 cells both in vitro and in vivo and show a potential beneficial effect in suppressing the growth of hormone-dependent breast cancer. Several treatments with intraperitoneal injections support its anti-cancer activity on tumor cell growth in different cancer models, such as the orthotopic model of breast cancer in athymic nude mice [100] and human hepatocellular carcinoma tumor growth [101]. Similarly, inhibitory activities on proliferation [104] and metastasis by inactivating STAT3 [101] have also been reported.

Along with the anticancer effects, some protective effects against deleterious effects of chemotherapeutic drugs on health cell models have also been reported. Ayoub et al. [104] explored the effect of (−)-oleocanthal treatment on growth of luminal breast cancer cells (BT-474, MCF-7 and T-47D) combined with tamoxifen. Results showed that (−)-oleocanthal inhibits growth of BT-474, MCF-7 and T-47D human breast cancer cells in mitogen-free media. Combined (−)-oleocanthal and tamoxifen treatments result in a synergistic growth inhibition of all cells. As was suggested for HT, these changes could also be present when this compound is orally administrated, although further studies are needed to prove this hypothesis.

### 2.4. Oleuropein and Oleuropein Aglycone

Oleuropein (OLE) is an ester of hydroxytyrosol (3,4-DHPEA) and the elenolic acid glucoside [46]. OLE belongs to a specific group of coumarin-like compounds, the secoiridoids that are glycosidically bound and produced from the secondary metabolism of terpenes. This compound has shown in vivo to exert preventive effects against early steps of cancer onset. In an azoxymethane (AOM)-induced mouse model of colerectal cancer, OLE has shown to reduce tumor incidence from 57 to 14% in the medial colon segment, which is related to the prevention of DNA damage (i.e., mutations) by the carcinogen [105] (Table 3). Concerning anti-cancer activity, previous studies have shown that OLE inhibits cell proliferation and induces apoptosis in breast, colorectal and thyroid cancer [106,107,108]. More recently, Yan et al. [109] have investigated the OLE effects on hepatocellular carcinoma cells, finding a reduction of HepG2 cell viability, as well as apoptosis induction in a dose-dependent manner through activation of the caspase pathway. This effect was correlated with ROS generation that could be responsible of both effects. Additionally, changes in proapoptotic Bcl-2 family members (BAX and Bcl-2) and inhibition of PI3K/Akt signaling pathway were reported to be involved in OLE-induced apoptosis, which could be ROS-dependent, at least in part.

On the other hand, OLE aglycone presents a similar structurebut with EA it is in the agliconed form, instead of the glycosydized form.Secoiridoids of VOO in aglyconic forms arise from glycosides in olive fruits by hydrolysis of endogenous β-glucosidases during crushing and malaxation [111]. First studies supporting the potential use of OLE aglycone as a nutraceutical in relation to cardiovascular health was provided, owing to the anti-inflammatory activities observed in a carrageenan-challenged mouse model of inflammation [112]. In addition, OLE aglycone inhibits TNFα-induced MMP-9 in a monocyte cell line, which could also been observed against other events induced by pro-inflammatory stimuli [113]. In more recent years (Table 4), OLE-aglycone has shown to be able to interfere with transthyretin (TTR) aggregation and to protect mouse HL-1 cardiomyocyte cells against the cytotoxicity induced by previously grown amyloid aggregates of wild-type TTR and L55P-TTR [110]. The results indicated that OLE-aglycone reduces amyloid TTR toxicity, specifically to a cardiac cell line.

### 2.5. Others Minor Compounds

In the last few years, other additional compounds found in VOO or EVOO, but in less amount, have been tested to determine their potential for health maintenance. Thus far, oleanolic acid and maslinic acid are known to have beneficial properties against different types of cancers [114,117,118,119,120,121,122,123,124]. Recently, Sánchez-Quesada et al. [114] sought to elucidate whether both compounds possessed chemopreventive activity on two cell lines of human breast cancer cells (MDA-MB-231 and MCF7) (Table 4). The results showed that oleanolic acid inhibits the proliferation and causes oxidative stress of highly invasive cells. Other two triterpenic dialcohols, uvaol and erythrodiol, have been reported to present anti-tumoral effects in leukemic cells, in skin mice tumors and in astrocytoma cells [117,125,126,127], but there is no evidence about their effects in highly invasive human breast cancer cells. For this purpose, Sánchez-Quesada et al. [115] have evaluated their effects on cell proliferation, viability, cell cycle profile, apoptotic induction, oxidative stress and DNA oxidative damage in MDA-MB-231. Results showed that uvaol protects DNA from damage, whereas erythrodiol enhances damage to DNA. On the other hand, Lamy et al. [77] also reported that taxifolin is a strong inhibitor of in vitroangiogenesis by the inhibitory effect on specific autophosphorylation sites of VEGFR-2. Finally, Corominas-Faja et al. [116] showed that the secoiridoid decarboxymethyl OLE aglycone (DOA) blocks the multicellular tumorspheres formation from single-founder stem-like breast cancer cells. Moreover, mice orthotopically injected with CSC-enriched breast cancer-cell populations remain more time without cancer if the cells are pretreated with DOA, supporting the capability of this compound to reduce tumor-forming capacity. In addition, it has been evidenced that these effects could be due, at least in part, to a dual activity as an mTOR/DNA methyltransferase inhibitor, which can suppress CSC-like states responsible for maintaining tumor-initiating cells [116]. The election of this interesting compound is based on an phenotypic drug discovery in silico approach coupled to mechanism-of-action profiling and target deconvolution, identifying DOA, among the different phenolic components of EVOO, as an interesting compound capable to selectively target subpopulations of CSC with typical functional traits in breast cancer.

## 3. Conclusions

The present work aimed to explore the effects of the main phenolic compounds isolated from OO on different cancer or CVD aspects in studies published during the last five years. During this period, the potential preventive or therapeutic properties of phenolic compounds have been studied with special emphasis in cancer.

HT reduced cellular viability by means of different mechanisms including oxidative stress promotion and the PI3K/Akt pathway stimulation. Moreover, it induced apoptosis and arrest of a cellular cycle, leading to a cell proliferation reduction. An inhibitory action on specific autophosphorylation sites of VEGFR-2 was also reported, leading to the inhibition of endothelial cell signaling with a reduction of angiogenesis.

With respect to oleocanthal, it reduced cellular viability by lysosomal membrane permeabilization and/or overstimulation of autophagy initiation, processes that would lead to cell death. As for HT, it promoted the arrest of cell cycle, reducing therefore the cellular proliferation. In some cell lines, this effect was also due to their action in the estrogen receptors. Moreover, it suppressed HFG-induced activation of STAT3, which prevent malignant cell migration and invasiveness.

Finally, OLE showed a reduction of the DNA oxidation as a consequence of the treatment with some carcinogens. This would delay or prevent the carcinogenesis process initiation.

Overall, these results would indicate that HT and oleocanthal presented promising effects in vitro and in vivo studies, suggesting their potential as nutraceuticals for the prevention and management of NCDs.

## Figures and Tables

**Table 1:** Studies on hydroxytyrosol (HT) and derivatives effects on cancer and cardiovascular diseases (CVDs) in the last five years.

Sample	Treatments	Main Results	Ref.
CRC adenocarcinoma cells (DLD1)	HT (0–300 µM for 24 and 48 h)	HT induces ROS generation and leads to PI3K/Akt pathway activation, decreasing the antioxidant defense capacity through FOXO3a suppression.	[71]
Human CCA (TFK-1 and KMBC) and human gallbladder (GBS-SD) cancer cells	HT (0–200 μM for 24, 48 and 72 h)	HT induces cell cycle arrest and apoptosis.	[72]
Human hepatocellular carcinoma (HepG2, Hep3B, SK-HEP-1 and Huh-7) cells	HT (0–400 µM for 48 and 72 h)	HT can suppress the activation of Akt and NF-κB pathways.	[73]
Human breast (MDA and MCF-7), prostate (LNCap and PC3) and colon (SW480 and HCT116) cancer cells	HT (100 µM for 24, 48, 72, 96, 120 and 144 h)	HT inhibits the proliferation of all cell lines.	[74]
Human thyroid carcinoma (TPC-1 and FB-2), papillary and follicular (WRO) cells	HT (65–973 μM for 24 and 48 h)	HT reduces viability in all cell lines and exerts proapoptotic effects on papillary and follicular cancer cells.	[75]
Human colon cancer cells (Caco-2 and HT-29)	HT (100–200 μM for 8 and 48 h)	HT produces cell cycle arrest and promotes apoptosis.	[76]
Human umbilical vein endothelial cells (HUVECs) and dermal microvascular endothelial cells (HMVECs-d-Ad)	HT (0–50 μM for 18 and 24 h)	HT inhibits VEGFR-2 signaling pathway.	[77]
Male nude BALB/c mice cholangiocarcinoma xenograft (6–8 weeks old)	Intraperitoneally injected HT (500 mg/kg, daily, 3 weeks after the tumor volume reached ~120 mm^3^)	HT inhibits cholangiocarcinoma growth.	[72]
Orthotopic HCC model in nude mice cholangiocarcinoma xenograf (4–6 weeks old)	Intraperitoneally injected HT (10 mg/kg or 20 mg/kg, daily, 3 weeks starting 14 d after inoculation)	HT inhibits cholangiocarcinoma growth.	[73]
Human colon cancer (Caco-2) cells	HT (50 μM for 24 h)	HT up-regulates *CNR1* gene via epigenetic regulation (decrease in methylation at *CNR1* promoter), which is associated with reduced proliferation of Caco-2 cells.	[79]
Murine pre-adipocytes (3T3-L1) exposed to H_2_O_2_	Pretreatment with HT (1 and 5 µM for 24 h)	HT blunts the H_2_O_2_-induced GSH/GSSG alteration.	[84]
Human umbilical cord vein endothelial cells (HUVEC)	HT, Tyr, and combination of both (10 µM for 30 min or 18 h)	The combination of HT with Tyr preserves cell functions from oxidative damage, which correlates with rescuing their antioxidant properties.	[85]
Human myelomonocytic cells (U937) and murine skeletal myoblasts (C2C12) exposed to H_2_O_2_	Pretreatment with Laur-HT (5 µM), HT (20 µM) or both combined (20 µM) (for 30 min)	Laur-HyT has a protective antioxidant effect against H_2_O_2_ treatment, greater than HyT, so having a role in the prevention of apoptotic death in normal and tumor cells.	[86]
Human endothelial cells (ECV304) incubated with high glucose (30 mM) in the presence or absence of 0–120 mM FFAs (oleic or linoleic acid)	Co-treatment with HT (10 µM for 48 h) and polyphenol extract from EVOO (10 µM gallic acid equivalents for 48 h)	Treatments reduce the oxidative stress and modulate changes in NO and ET-1 associated with experimental conditions that simulate diabetes (hyperglycemia and a high level of FFA).	[87]
Human peripheral blood mononuclear cells (PBMC) and U937 monocytes activated with PMA (30 nM)	HT (1–10 μM for 0–24 h) prior to activation with PMA	HT blunts monocyte matrix invasive potential, reduces MMP-9 release and expression, and inhibits PGE2 production and COX-2 expression, which are mediated by inhibition of NF-κB transcription, PKCα and β1 activation.	[83]
Healthy subjects (22–37 years)	HT-enriched biscuits (30 g that contained 5.25 mg of HT) or Non-enriched biscuits (30 g) after overnight-fasting, only one meal in a cross-overdesing	Enriched biscuits consumption leads to a peak of posprandial levels of plasma metabolites (mainly 3,4-dihydroxyphenylacetic acid (DOPAC)-sulphate, DOPAC, HVA) between 0.5 and 1 h, which are also extensively excreted in urine and lower postprandial ox-LDL levels.	[88]
Volunteers with mild hyperlipidemia	HT purified (99.5%) from olive mill waste (5 mg, daily, for 8 weeks)	HT does not influence markers of CVD, blood lipids, inflammatory markers, liver or kidney functions and the electrolyte balance, but increased vitamin C levels.	[89]
Human hepatocarcinoma (HepG2) cells under tunicamycin-induced ER stress	HT or hepatic HT-derived metabolites 3-*O*-HT glucuronide and 4-*O*-HT glucuronide (10 and 25 μM for 24 h) prior to tunicamycin treatment	Both metabolites glucuronide inhibit ER stress, although they induce a milder change in mRNA expression levels of both CHOP and BiP.	[90]

**Table 2:** Studies on (−)-oleocanthal effects on cancer and CVDs in the last five years.

Sample	Treatments	Main Results	Ref.
Human breast cancer cells (MDA-MB-231, MCF-7 and BT-474)	(−)-Oleocanthal (10–100 ng/mL for 24, 48 and 72 h)	(−)-Oleocanthal inhibits growth and causes a dose-dependent inhibition of HGF-induced cell migration, invasion and G1/S cell cycle progression.	[100]
Human pancreatic (BxPC3), prostate (PC3) and breast (MDA-MB-231) cancer cells	(−)-Oleocanthal (0.2–20 µM for 4, 24, 48 and 72 h)	(−)-Oleocanthal induces cell death, primary necrotic and apoptotic cell death via induction of lysosomal membrane permeabilization.	[102]
Human breast cancer (MCF-7, T47D) metastatic breast cancer (MDA-MB-2318), CRC (Caco-2) and adenocarcinoma (HeLa) cells	(−)-Oleocanthal (10 μM for 72 h on MDA-MB-231)	(−)-Oleocanthal shows anti-proliferative against several breast cancer cell lines and down-regulates the levels of p-mTOR in the metastatic breast cancer cell line (MDA-MB-231).	[103]
Human hepatocellular cell lines (Huh-7, HepG2 and HCCLM3)	(−)-Oleocanthal (0–80 µM for 12, 24, 48 and 72 h)	(−)-Oleocanthal inhibits human hepatocellular carcinoma by inactivating STAT3.	[101]
Human breast cancer cells (BT-474, MCF-7 and T-47D)	(−)-Oleocanthal (5–60 µM for 48 h in BT-474 and MCF-7 cells; 10–100 µM for 24 and 48 h in T-47D cells)	(−)-Oleocanthal suppresses growth of all cancer cells, in part, by reducing total levels of ERα.	[104]
Female athymic nude Foxn1^nu^/Foxn1^+^ mice (4–5 weeks old) in human tumor xenograft model	Intraperitoneally injected (−)-oleocanthal (5 mg/kg, 3 d/week, 33 d starting 5 d after inoculation)	(−)-Oleocanthal suppresses tumor growth.	[100]
BALB/c athymic nude mice a in vivo human lung metastasis model hepatocellular (4–6 weeks old, male)	Intraperitoneally injected (−)-oleocanthal (5 mg/kg or 10 mg/kg, daily, 5 weeks)	(−)-Oleocanthal suppresses hepatocellular tumor growth and impedes carcinoma metastasis in lung by inactivating STAT3.	[101]
Female thymic nudeFoxn1^nu^/Foxn1^+^ mice (4–5 weeks old) inoculated with BT-474 cells	Intraperitoneally injected (−)-oleocanthal (5 mg/kg per d or 10 mg/kg, 3 d/week, 43 d)	(−)-Oleocanthal reduces total levels of estrogen receptors in BT-474 cells.	[104]

**Table 3:** Studies on OLE (Oleuropein) and OLE aglycone effects on cancer and CVDs in the last five years.

Sample	Treatments	Main Results	Ref.
Human umbilical vein endothelial cells (HUVECs) and dermal microvascular endothelial cells (HMVECs-d-Ad)	OLE (0–50 μM for 18 and 24 h)	OLE does not inhibit VEGFR-2 signaling pathway.	[77]
Mice with colon cancer induced by AOM injections (10 mg/kg, 1 d/week for 6 weeks)	Basal diet either enriched or not with OLE (125 mg/kg), (7 or 17 weeks)	OLE-enriched diet prevents the preneoplastic lesions in different colon segments, reducing the severity of crypt dysplasia and DNA damage in peripheral leukocytes.	[105]
Mouse atrial myocytes (HL-1)	OLE-aglycone (60 μM for 24 h)	Data suggest a possible use of OLE-aglycone to treat human transthyretin (TTR)-related pathologies with the aim to relieve or to delay the occurrence of the most severe cardiac symptoms.	[110]
Luminal MCF-7 breast cancer cell	OLE (100 μM or 200 μM for 72 h)	OLE-induced apoptosis, which is associated with Bax gene expression up-regulation and Bcl2 gene expression down-regulation via p53 pathway activation.	[106]
Thyroid tumorTPC-1 and BCPAP cells	OLE and Ac-OLE (10, 50, and 100 mM for 48 h)	Both treatments inhibit cell proliferation, and decrease H_2_O_2_-induced ROS levels, and p-Akt and p-ERK levels. Thus, it exerts antioxidant and inhibitory effects on growth-promoting signal pathways.	[107]
Human colon adenocarcinoma (HT-29) cells	OLE (0 μM–800 μM for 24, 48 and 72 h)	OLE inhibits cell growth and induces apoptosis, which is associated with a decrease in HIF-1α protein and an increase p53, but not to changes in IkB-α and MAPK cascade proteins.	[108]
Hepatocellular carcinoma (Huh7) and human hepatoma (HepG2) cells	OLE (0, 20, 40, 60, 80 or 100 μM for 24 h)	OLE induces apoptosis in HepG2 cells in a dose-dependent manner, via caspase activation which is mediated by changes in proapoptotic Bcl-2 family members, (BAX and Bcl-2) levels, down-regulation of PI3K/AKT signaling pathway, and ROS production increases.	[109]

**Table 4:** Studies on other minor compound effects on cancer and CVDs in the last five years.

Sample	Treatments	Main Results	Ref.
Human umbilical vein endothelial cells (HUVECs) and dermal microvascular endothelial cells (HMVECs-d-Ad)	Taxifolin (0–50 μM for 18 and 24 h)	Taxifolin inhibits VEGFR-2 signaling pathway.	[77]
Human breast cancer cells (MDA-MB-231 and MCF7)	AO and MA (0.001–100 μM for 4, 24, 48 and 72 h)	AO inhibits the proliferation and increases the oxidative stress of highly invasive cells.	[114]
Invasive human breast cancer cells (MDA-MB-231)	UV and ER (0. 001–100 µM for 4, 24, 48 and 72 h)	UV protects DNA from damage, whereas ER enhances damage to DNA.	[115]
SUM-159 cells subcutaneously injected into athymic nude mice; or into the 2nd right mammary fat pad of female SCID/Beige mice	Pretreatment with DOA (10, 20 μmol/L for 3 d); or graded concentrations of DOA (for 2 h)	DOA blocks the formation of multicellular tumorspheres generated from single-founder stem-like cells in a panel of genetically diverse breast cancer models and suppresses CSC-like states responsible for maintaining tumor initiating cell properties within breast cancer populations.	[116]

## Abbreviations

AO	oleanolic acid
AOM	azoxymethane
COX-1	cyclooxygenase-1
COX-2	cyclooxygenase-2
CSC	cancer stem cell
CVDs	cardiovascular diseases
DOA	decarboxymethyl OLE aglycone
EA	elenolic acid
ECS	endocannabinoid system
ER	endoplasmic reticulum
ERK	extracellular signal-regulated kinase
ET-1	endothelin-1
EVOO	extra virgin olive oil
FFA	free fatty acid
GSH	glutathione
GSSG	oxidized glutathione
HGF	hepatocyte growth factor
HT	hydroxytyrosol
HUVEC	human umbilical cord vein endothelial
IL-1β	interleukin 1 beta
IL-10	interleukin-10
iNOS	inducible nitric oxide synthase
Laur-HT	hydroxytyrosyl laurate
LPS	lipopolysaccharide
MD	Mediterrean Diet
MMP	matrix metalloproteinase
MMP-9	matrix metalloproteinase 9
MMP-9/NGAL	neutrophil gelatinase-associated lipocalin complex
NF-κB	nuclear factor-kappa B
NO	nitric oxide
OLE	oleuropein
p-mTOR	phosphorylated mammalian target of rapamycin
PI3K	phosphoinositide 3-kinase
ROS	reactive oxygen species
TNFα	tumor necrosis factor α
TTR	transthyretin
Tyr	tyrosol
UV	uvaol
VEGF	vascular endothelial growth factor
VEGFR-2	vascular endothelial growth factor receptor-2
VOO	virgin olive oil
